# Influence of *COMT* (rs4680) and *DRD2* (rs1076560, rs1800497) Gene Polymorphisms on Safety and Efficacy of Methylphenidate Treatment in Children with Fetal Alcohol Spectrum Disorders

**DOI:** 10.3390/ijerph19084479

**Published:** 2022-04-08

**Authors:** Małgorzata Śmiarowska, Bogusław Brzuchalski, Elżbieta Grzywacz, Damian Malinowski, Anna Machoy-Mokrzyńska, Anna Pierzchlińska, Monika Białecka

**Affiliations:** 1 Department of Pharmacokinetics and Therapeutic Drug Monitoring, Pomeranian Medical University, Aleja Powstancόw Wielkopolskich 72 St., 70-111 Szczecin, Poland; malgorzata.smiarowska@wp.pl (M.Ś.); machalin@googlemail.com (B.B.); damian.malinowski@pum.edu.pl (D.M.); pierzchlinska@gmail.com (A.P.); 2 Department of Experimental and Clinical Pharmacology, Pomeranian Medical University, Aleja Powstancόw Wielkopolskich 72 St., 70-111 Szczecin, Poland; elagrzywacz@me.com (E.G.); anna.machoy.mokrzynska@pum.edu.pl (A.M.-M.)

**Keywords:** fetal alcohol spectrum disorders (FASD), attention deficit hyperactivity disorders (ADHD), methylphenidate treatment, catechol-O-methyltransferase (*COMT*), dopamine-2 receptor (*DRD2*), gene polymorphisms

## Abstract

Fetal alcohol spectrum disorders (FASD) in a course of high prenatal alcohol exposure (hPAE) are among the most common causes of developmental disorders. The main reason for pharmacological treatment of FASD children is attention deficit hyperactivity disorder (ADHD), and methylphenidate (MPH) is the drug of choice. The aim of the study was to assess whether children born of hPAE with ADHD, with or without morphological FASD, differ in terms of catechol-O-methyltransferase (*COMT*) and dopamine receptor D2 (*DRD2*) gene polymorphisms, and if genetic predisposition affects response and safety of MPH treatment. The polymorphisms of *COMT* (rs4680) and *DRD2* (rs1076560, rs1800497) were analyzed in DNA samples. A borderline significance was found for the correlation between MPH side effects and the G allele of *COMT* (rs4680) (*p* = 0.04994) in all ADHD children. No effect of *COMT* (rs4680) and *DRD2* (rs1076560, rs1800497) polymorphisms and the treatment efficacy was observed. The analyzed *DRD2* and *COMT* gene polymorphisms seem to play no role in MPH efficacy in ADHD children with hPAE, while low-activity *COMT* (Met158) variant carriers may be more intolerant to MPH. The MPH treatment is effective in ADHD independent of FASD, although the ADHD-FASD variant requires higher doses to be successful. These results may help in optimization and individualization in child psychiatry.

## 1. Introduction

Alcohol consumption during pregnancy may lead to permanent damage in the offspring, resulting in, for example, Fetal Alcohol Spectrum Disorders (FASD). FASD include Fetal Alcohol Syndrome (FAS), partial Fetal Alcohol Syndrome (pFAS), alcohol-related neurodevelopmental disorders (ARND), and alcohol-related birth defects (ARBD) [1]. FAS is believed to be the most common teratogenically induced, nonhereditary form of mental deficiency throughout the Western world [2,3,4]. It is associated with reduced intelligence, attention disorders, neuropsychological deficits, physical abnormalities including facial dysmorphology, sleep disorders, and behavioral problems [5,6,7].

According to worldwide studies, alcohol consumption among women of childbearing age is increasing. At the same time, a large proportion of pregnancies are unplanned, which leads to more frequent cases of prenatal alcohol exposure (PAE) which may be explained by harmful and sustained alcohol intake during any trimester of pregnancy which, in consequence, may produce morphological, biochemical, and epigenetic (among others) consequences in the fetus. Alcohol has a prolonged effect in pregnancy in terms of abundance and efficiency caused by the differences in maternal and fetal enzymes, oxidative stress/reactive oxidative species (ROS) and, additionally, reduced elimination. In 120 min after the mother’s alcohol intoxication, its concentration in her blood increases to maximum (blood alcohol content BAC). Owing to placenta transport of alcohol, its concentration after 120 min gets maximal BAC which is nearly the same as maternal. During pregnancy there is no safe dose of alcohol and most of PAE state in the first trimester of pregnancy [8]. The structural and neurobehavioral consequences associated with PAE are the most teratogenic in the brain. Post-mortem and brain imaging studies showed reductions and anomalies in the brain size and shape (seen as microcephaly and hydrocephalus), especially in the cerebellum, basal ganglia, and corpus callosum, as well as grey matter heterotopia and neurochemical abnormalities [9,10].

The multiple clinical consequences of the prefrontal cortex area damage in FASD children involve cognitive domains (general intelligence, visual perception and construction and learning and memory), motor functions and activity levels, language skills and adaptive functioning [11,12,13,14]. In addition, PAE has a high comorbidity rate with other learning and behavioral disorders [15]. The attention deficit hyperactivity disorder (ADHD) is one of them [16,17,18,19]. A correlation between FASD and ADHD has become a focus of special scientific attention. It was established that FASD constitutes one of the main susceptibility factors for developing ADHD (relative risk 7.6; attributable risk 86.8%). Similarly, displaying the symptoms of ADHD increases the probability of diagnosing FASD (relative risk 13.28; attributable risk 92.5%) [20]. There are numerous, likely interacting, genetic and environmental factors involved in mediating ADHD and its severity [21,22,23]. Since FASD and ADHD are characterized by similar phenotype expression, prevalence, and detrimental impact they have on the child’s development, a better understanding of neurobiological underpinnings of PAE-related ADHD may lead to a faster diagnosis and more effective pharmacological treatment of ADHD symptoms [24,25].

In addition to psychological and behavioral therapies, NICE guidelines (National Institute for Health and Care Excellence) allow a pharmacological treatment of individuals older than 5 years with a severe presentation of ADHD. Stimulants, such as methylphenidate (MPH), are considered to be the most effective medications for the improvement in ADHD symptoms and MPH is often the first-choice substance owing to its effectiveness and safety, as demonstrated in several studies [26,27].

The mechanism by means of which MPH reduces symptoms in ADHD is not completely clear. One of the most known conceptions of the ADHD pathogenesis is the one related to delayed dopaminergic system maturation in the brain, and medication such as MPH can improve DA transmission. It seems to be similar to cocaine and involved in the central stimulation of catecholamines. It is believed that MPH binds to dopamine transporter (DAT) and norepinephrine transporter (NET) in the presynaptic membrane. In this way, it blocks the reuptake of dopamine and norepinephrine and in turn increases intrasynaptic concentrations of DA dopamine and norepinephrine (NE) and increases them in the synaptic cleft [28]. Depending on the brain region activation, these catecholamines (especially DA) have a specific clinical manifestation; for example, in the prefrontal cortex (PFC) they are more responsible for cognitive control–executive functions (inattentive type of patients), while in the subcortical brain regions (a striatal–frontal loop) they are more strongly associated with motivation and reward (the hyperactive and combined types) [28]. The other mechanism by means of which MPH affects the hyperactivity and emotional disturbances (aggression and anxiety) in ADHD is probably connected with its indirect stimulation of the dopamine D2 postsynaptic receptor (DR2) [29,30,31]. PFC contributes to the unusual property of the DA system, and there is a hypothesis of exceptional PFC’s vulnerability to environmental and genetic variation [32]. As mentioned before, not only environmental but also genetic factors may be involved in ADHD etiology and its severity. Many studies have extensively investigated the role of polymorphisms in the DR2 gene (*DRD2*), e.g., Taq1A (rs1800497), C957T (rs6277), and −141Cins/del (rs1799732), in neuropsychiatric disorders such as schizophrenia, alcoholism, Parkinson’s disease, substance addiction, and ADHD [33,34,35,36,37,38]. A meta-analysis conducted by Wu et al. identified a significant association between ADHD and Taq1A polymorphism of *DRD2* (OR 1.65, 95% CI 1.05–2.58, *p* < 0.0001) [39]. The result was supported by a subsequent meta-analysis (95% CI = 1.068–2.984, *p* = 0.027) [38]. Interestingly, the authors observed differences between ethnicities: the polymorphism was associated with ADHD in Africans, but not in East Asians or Caucasians. Aggressive behavior, which is very close to impulsiveness, is one of the clinical symptoms met in ADHD. The study conducted by Zai at al. explored the role of dopaminergic system genes in the etiology of aggressive behavior in children [40]. The authors discovered a significant association between A-241G (rs1799978), rs1079598, and Taq1A (rs1800497) of *DRD2* polymorphisms and childhood aggression. Genetic variation in enzymes that act on the biosynthesis or degradation of DA and its metabolites may influence the effectiveness of MPH in ADHD treatment [41]. Catechol-O-methyltransferase (*COMT*) inactivates biologically active or toxic catecholamines such as DA and norepinephrine (NE) and plays a role in the metabolism of drugs (L-dopa). Differences in *COMT* activity and genotype may determine individual variations in the therapeutic response to drugs, whose mechanism of action are linked to DA and NE levels. A single *COMT* gene, located on chromosome 22q11, encodes both the acid-soluble (S-*COMT*) and membrane-bound (MB-*COMT*) forms of this enzyme. The most studied polymorphism of the *COMT* gene is a G to A transition at codon 158 [Val158Met; rs4680]. The transition resulting in the substitution of methionine for valine has been linked to low *COMT* enzyme activity and is designated as the L (low-activity) allele, in contrast to the H (high-activity) allele [42].

Jung et al. found a correlation between the *COMT* Val158Met polymorphism and cortical thickness and surface area in children with ADHD, cortico-cerebellar executive function network, or stimulants’ efficacy [43]. The impact of genetic factors on ADHD pathogenesis, as well as on the efficacy and safety of MPH treatment in ADHD patients, has been analyzed, however, without any conclusive results [44,45,46,47]. The study aimed at assessing whether children with ADHD, with or without FASD, differ in terms of catechol-O-methyltransferase (*COMT*, rs4680) and dopamine receptor D2 (*DRD2*, rs1076560 rs1800497) gene polymorphisms, and if the genetic predisposition affects the response and safety of the methylphenidate (MPH) treatment in children with ADHD and high prenatal alcohol exposure (hPAE) with or without morphological features FASD, according to the Washington Questionnaire.

## 2. Materials and Methods

### 2.1. Study Group

**Figure 1:**
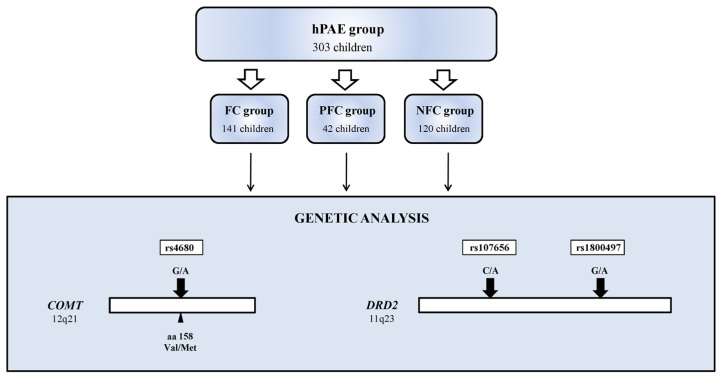
The flowchart of the study design (hPAE—high prenatal alcohol exposure, FC—FAS children, PFC—partial FAS children, NFC—no FASD children).

Three hundred and three children were included in the study: 213 girls (mean age 8.59 ± 4.77 years) and 90 boys (mean age 9.77 ± 5.14 years). The study group consisted of 303 hPAE children, with 183 ones diagnosed with FASD (partial FAS children—PFC, *n* = 42, and FAS children—FC, *n* = 141) and NFC children without the presence of evident morphological FAS changes unless positive history of hPAE (non-morphological FAS children—NFC, *n* = 120) (Table 1, Figure 1). All children enrolled in the study met the criteria for the occurrence of high alcohol exposure during fetal life/(hPAE). PAE was confirmed by the mother’s medical history or by objectively documented background survey (e.g., mother’s stay in a sobering station or in a detoxification ward during pregnancy). The clinical diagnosis of FASD was based on a 4-digit diagnostic questionnaire according to a validated validation of the Polish version of the Washington Questionnaire for the Assessment of Fetal Alcohol Spectrum Disorders [48]. It is worth pointing out that all of FASD children enrolled to the present study had severe OUN lesions or disturbed functionality which were not related to genetic or environmental factors (both FC and PFC received 3 or 4 points on the 4th degree scale) and they were short-statured or had specific face anomalies. They are generally moderate (3rd and 2nd degree) ARND according to the Washington Questionnaire.

In the total of 303 hPAE children enrolled for the study, 114 children met the criteria for ADHD diagnosis according to the American Psychiatric Association (APA) DSM-IV criteria [49] and the ICD-10 classification of Mental and Behavioral Disorders: Clinical Description and Diagnostic Guidelines [50]. The quantitative assessment of the severity of symptoms was performed according to the questionnaire developed by Wolańczyk and Kołakowski based on the DSM-IV rating scale (RS) and ICD-10 criteria which is not an international diagnostic valid instrument for ADHD screening, but a very approachable and popular form of interviewing and simple in its interpretation form in common practice (the pattern is available in Supplementary Materials) [51]. It examines three areas of the child’s dysfunction using separate scales: (I). attention deficit includes nine descriptions of problem behaviors, (II). hyperactivity—five, (III). impulsivity—four. The severity of each symptom is measured from 0 to 3 points (0—the symptom occurs never or very rarely, 1—sometimes, 2—often, 3—very often). Therefore, in each of the scales of the examined area, the patient may obtain a maximum of 27 in I scale, 15 in II scale, and 12 points in III scale, respectively. The numerical values served as a reference for the assessment of the intensity of attention deficit, hyperactivity, impulsivity before (BT) and after the treatment (AT) of MPH.

According to the DSM-IV RS criteria, the examined children were divided into 3 subgroups (subtypes) depending on the predominance of the clinical picture (attention deficit disorder vs. activity/impulsivity assessed together): attention deficit/hyperactivity disorder (ADHD-A), hyperactivity/hyperimpulsivity disorder (ADHD-HI), and a mixed form (ADHD-M) when criteria of both subtypes are balanced.

The ICD-10 classification was used to investigate the contribution of the child’s environmental functioning conditions to the clinical manifestation of hyperkinetic syndrome (F90.0/ADHD) symptoms to consider all probable factors playing a role in its pathogenesis, except biological pathways of catecholamines involved in it. According to the diagnosis criteria, F90.0 child’s impaired functioning includes at least 2 out of 3 areas examined, including peer group, school, and home, which are observed at the same time by minimum 2 out of 3 assessments from the perspective of various people from the child’s environment.

The criteria for the patient’s exclusion from the study were psychiatric disorders: psychotic (present and in medical history), significant mood and anxiety disorders requiring pharmacological treatment, psychoactive substance (SPA) addiction, risky and harmful use of SPA (present and in medical history); and developmental disorders: autism spectrum, profound and significant mental retardation, genetic diseases, severe and uncompensated somatic diseases (endocrinological, cardiovascular, renal, neoplastic, autoimmune, anorexia), and organic diseases with clinical manifestation (epilepsy). A slight shortage of weight and height according to age, but within normal limits (>10th centile), and a history of depressive episodes and emotional and behavioral disorders did not exclude patients from the study.

### 2.2. Eligibility for MPH Treatment

Within the study group, i.e., 303 children with hPAE, 114 children were included in the methylphenidate treatment. The children were older than 6 years old, with a diagnosis of ADHD, who did not benefit from cognitive behavioral therapy (CBT) for last 6 months. The 6 months psychotherapy condition did not apply to children whose ADHD symptoms were severe and caused dysfunctional environmental functioning. Before the introduction of the drug, each patient underwent examinations: electrocardiography, blood pressure, heart rate, weight, and height. Initial doses of 5 mg and 10 mg MPH in short-acting forms were used to assess drug tolerability (severity of nausea, abdominal pain, decreased appetite, and cardiovascular effects) and appropriateness of further administration. Established MPH treatment was continued with prolonged-release forms approved for use in Poland: Medikinet 10 CR (10 mg methylphenidate hydrochloride, by Medice) and Concerta 18 (18 mg methylphenidate, by Janssen). The doses were increased twofold: Medikinet 20 CR and Concerta 36 mg, which were the therapeutic doses and the basis for analysis in the current study. The maximum dose (Medikinet 60 CR and Concerta 54 mg) was used only in individual cases, which did not affect the general observation. The choice between Medikinet CR vs. Concerta was based on the patient’s daily requirement for adjustments and according to the drug’s half-life (T_T1/2_ = 8 h vs. T_T1/2_ = 12 h, respectively).

### 2.3. Assessment of Efficacy and Safety of MPH Treatment

The evaluation of the efficacy and tolerability of methylphenidate was carried out based on the data from the parents or guardians and other persons from the child’s functioning environment (teacher, guidance counselor, or educator in the institution). To measure the therapeutic effect of MPH, the questionnaires based on DSM-IV RS, described in the methodological section, were applied. The first assessment was performed at time “0” at the qualification for treatment. The treatment effect was assessed at three time points (after 8, 12, and 24 weeks), although the period of the first 8 weeks was the best to estimate the control exam to be sure that all children followed the established regimen. The results were effective when the number of problematic symptoms in terms of the examined scale (attention deficits, hyperactivity, and impulsivity, respectively) was reduced by more than 40% in at least two of the examined environments.

The assessment of MPH adverse effects (AEs) was based on the occurrence of symptoms such as: loss of appetite and weight loss (greater than 10% in 4 weeks), nausea, abdominal pain, headache, sleep problems, cardiovascular and emotional disorders (increased fear, anxiety, aggression and autoaggression), fatigue, and skin lesions. The criterion for discontinuation of the treatment due to AEs (according to the characteristics of the product) was the occurrence of any of the following symptoms: cardiac arrhythmia, appearance of suicidal thoughts or intentions, perceptual disturbances (visual, auditory, sensory hallucinations), attacks of uncontrolled aggression and agitation (which had not been present before the drug was started), motor and/or vocal tics, sudden increase in body temperature, or morphological changes in the blood picture (decrease in the cell count). No such severe adverse effects during MPH treatment appeared to exclude any patient from the observation.

Written consents were obtained from the legal guardians of all the studied children, and children who were 16 years of age or older. The study was approved by the local Bioethics Committee at the Pomeranian Medical University in Szczecin (the approval of the Bioethics Committee of the Pomeranian Medical University in Szczecin from 5 October 2015, No. KB-0012/95/15).

### 2.4. Genetic Analysis

Genomic DNA was extracted from buccal swab samples, and cells from mucus membrane were collected using a sterile synthetic swab. Prior to the isolation, swabs were stored in a preservative buffer at +4 °C. Extraction was performed using Genomic Micro AX SWAB Gravity kit (A&A Biotechnology, Gdynia, Poland) following manufacturer’s protocol. DNA was subsequently standardized to equal concentrations of 10 ng/μL, based on spectrophotometric absorbance measurement (260/280 nm). List of the SNP assays is shown in the table below (Table 2).

### 2.5. Genotyping

Genotyping for *COMT* rs4680, *DRD2* rs1076560, and rs1800497 SNPs was performed using pre-validated allelic discrimination TaqMan real-time PCR assays (Life Technologies, Waltham, MA, USA), and TaqMan GTXpress Master Mix (Life Technologies, Waltham, MA, USA). All reactions were run in a final volume of 12 μL (reaction temperature profile: 95 °C, 20 s, 40 × 95 °C 1 s/60 °C, 20 s).

Fluorescence data were captured using the ViiA7 Real-Time PCR System (Applied Biosystems, Waltham, MA, USA) after 40 reaction cycles. Specific genotypes were assigned to individual samples after the analysis with TaqMan Genotyper software (Thermo Fisher Scientific, Waltham, MA, USA).

### 2.6. Statistical Analysis

The chi2 (χ^2^–Pearson’s test) and/or Fisher’s exact test were used to compare qualitative variables between genotype groups, alleles, as well as clinical data in genotype groups. For genotype and allele distribution frequencies Hardy–Weinberg equilibrium was calculated. Age distribution according to SNP genotypes was performed using Kruskal–Wallis test. *p* < 0.05 was considered statistically significant. Statistical analysis was performed using STATISTICA PL, ver. 13.1 software (StatSoft, Inc. 2016, Tulsa, OK, USA, STATISTICA-data analysis software system, version 13.1) software. Haplotype reconstruction and linkage disequilibrium analysis were performed by means of HaploView 4.2 software.

## 3. Results

In the group of 303 children with hPAE enrolled for the study, 114 were diagnosed with ADHD. Seventy-one of them had morphological symptoms of FASD (partial FAS children—PFC and FAS children—FC) and 43 children did not show any morphological changes of FASD (no FASD children—NFC). The clinical picture of children with ADHD included features of attention deficit disorder and hyperactivity with attention deficit (ADHD-A), and attention deficit disorder and hyperactivity with predominantly hyperactivity and impulsivity (ADHD-HI). No individuals were diagnosed with the mixed form of ADHD (ADHD-M).

According to the protocol, MPH was introduced in 114 children with ADHD symptoms. The mean daily dose of MPH was 27.37 mg. However, in the FASD subgroup the established doses were significantly higher compared to the NFC subgroup (*n* = 71, dose 31.6 mg vs. *n* = 43, dose 18.67 mg, respectively, *p* = 0.0112).

In both the FASD/ADHD and NFC/ADHD groups, pharmacological treatment was effective in >90% of the patients (*n* = 104 successfully treated patients). No improvement was found in three patients treated with MPH, and in seven children, adverse effects (AEs) occurred, and the experiment was discontinued.

The efficacy of MPH among FASD/ADHD children was observed in the decreased symptoms of hyperactivity and impulsivity (*p* < 0.0001), with no improvement in attention deficits (*p* = 0.2024). In contrast, NFC/ADHD children showed statistically significant improvement in attention and reduction in hyperactivity (*p* < 0.001 and *p* = 0.0163, respectively) (Table 3).

Adverse side effects occurred in 7 out of 114 patients treated with MPH. Symptoms of intolerance appeared quickly after the drug introduction and were associated with noradrenergic and dopaminergic overstimulation (transient tachycardia, loss of appetite, headache, stomach pain, insomnia trouble, anxiety, and rise in autoaggression and aggression). Their clinical presentation and severity decreased after introduction of a modified form of MPH. No cardiotoxic effects or life-threatening symptoms were reported which could force discontinuation of the therapy in this group.

No associations were noted for the analyzed gene polymorphisms. *p*-values for allele frequency comparison and odds ratio (OR) for different inheritance models are given in Table 4 and Table 5, while full genotyping data is provided in Supplementary Table S1. The distribution of genotypes in accordance with the Hardy–Weinberg equilibrium was observed in the case of all studied SNPs. Separate subgroup analysis within the FASD group (FAS vs. NFC, pFAS vs. NFC, FAS + pFAS vs. NFC) show no significance (data not shown).

Considering linkage disequilibria between SNPs located within the same gene, the frequencies of haplotypes reconstituted from genotyping results for two *DRD2* SNPs (rs1076560 and rs1800497) were compared between the FASD and NFC groups. A-A and A-C *DRD2* haplotypes were less frequent among NFC children compared to the FASD (0.154 vs. 0.183, and 0.013 vs. 0.028, not significant), while the major G-C haplotype was more frequent among NFC children (0.829 vs. 0.784, not significant). Linkage disequilibria and haplotype frequencies are provided in Supplementary Tables S2 and S3.

In children without morphological signs of FASD who carried the minor A allele of the *COMT* rs4680:G > A polymorphism, the clinical picture was more often associated with emotional anxiety and/or depressive disorders, and MPH was the drug of choice. The mean age in this group was 8.45 years (SD = 3.56 years) and the mean dose used was 11.34 mg/d.

There was a statistically significant association between the occurrence of adverse reactions in children treated with MPH and the presence of the *COMT* rs4680 minor A allele (*p* < 0.049).

In 7 out of 114 MPH-treated children with ADHD, significant adverse effects occurred at the time of drug introduction, such as transient tachycardia, loss of appetite, headache, stomach pain, insomnia trouble, anxiety, and a rise in autoaggression and aggression. These symptoms, due to their clinical presentation and severity, decreased after the introduction of a modified form of MPH (Table 6).

Among the patients who developed side effects during the MPH treatment, there were a heterozygote and five AA homozygotes. Among carriers of the homozygous GG variant, no children were found to react negatively to MPH. The mean age of patients in our study who experienced the side effects was 8.45 years (SD = 3.56), and the ratio of girls to boys was 4:2. All those children belonged to the NFC group. The mean MPH dose in this group exceeded 10 mg/d (11.34 mg/d), mainly due to the form of the drug, having 18 mg/d as a starting dose.

No association of the studied polymorphisms: *DRD2* rs1076560:C > A or *DRD2* rs1800497:G > A with the efficacy or safety of MPH treatment was observed (Table 7).

## 4. Discussion

In the present study, we analyzed a possible relationship between *COMT* and *DR2* receptor gene polymorphisms and MPH treatment efficacy and safety in FASD children with ADHD symptoms. We demonstrated a positive impact of MPH on the reduction in ADHD symptoms in children exposed to hPAE, both with and without clinical signs of FASD. The effects of MPH treatment in ADHD groups, both in children with and without morphological features of FASD, were significant (FASD and NFC, *p* = 0.001 and *p* < 0.0001; properly, Table 3). Although, in the first group of FASD/ADHD children, higher MPH doses were required and they were more effective for hyperactivity and impulsivity features compared to the NFC/ADHD group, with a better response in cognition function and impulsivity. The authors made no comparison of these data to the sex of the examined children as it would have shown if there had been a relationship to clinical manifestation in both sexes. The scanty population under study was a real limitation to statistical analyses and this is going to be the next step of our research on hPAE risk in ADHD children. At the same time, in each subgroup, the pharmacological treatment led to a reduction in problematic symptoms but, importantly, in different areas. It was shown that the effectiveness of MPH was greater in the FASD group of children compared to the NFC group in reducing impulsivity and hyperactivity (*p* < 0.0001). On the other hand, there was a significant improvement in attention deficits in the NFC group (*p* = 0.001), which was not observed in the FASD group (*p* = 0.2024). These findings are consistent with the results of previously published studies [52,53,54]. A network meta-analysis of double-blind, randomized controlled trials, which included data of more than 10,000 children and adolescents, showed efficacy of MPH in the reduction in ADHD symptoms, which, in turn, confirmed its role as the first-choice medication for the short-term treatment of ADHD. However, the study did not conduct an in-depth analysis of the causes of ADHD, including the impact of hPAE, although it has been noted that individuals with FASD may respond differently to MPH than other children with ADHD [55]. Ter-Stepanian et al. examined the clinical response to MPH in 267 children diagnosed with ADHD, experiencing comorbid psychiatric disorders [52]. The authors showed that the presence of conduct disorder (CD) or oppositional defiant (ODD) was associated with good response to MPH, whereas children diagnosed with only comorbid anxiety were less likely to benefit from the treatment, independently of sex, age, or socioeconomic status. It may lead to a conclusion that when hyperactivity and impulsivity are dominant disturbances in “nasty” children/youth with conduct disorder, they may suggest a comorbidity of FASD and ADHD syndrome. Such observations could help with the aimed treatment procedures, as, so far, MPH has not been the first-choice medicament in CD because of the drug’s addiction risk opposite to ADHD.

On the other hand, MPH seems to be not so effective in individuals experiencing specific school skills disturbances with cognitive function disabilities as might have been suspected. Our observations confirm that children with an early ADHD onset in the course of FASD show executive deficits in several cognitive domains, e.g., verbal working memory, inhibitory control or planning, and reward regulation. Of particular interest are inattentive symptoms, which may negatively influence already existing disabilities and increase the risk of secondary disabilities. There is little evidence on the management of ADHD in FASD; thus, the recommendations are based on the assumption that MPH should be the first-line pharmacotherapy for patients with ADHD, despite the fact that 35% of the patients treated with MPH either do not respond to treatment or present adverse effects [56,57]. The results of our study may also lead to a conclusion that the necessity of a more detailed interview of hPAE history and clinical features of FASD during ADHD patient examination might be very helpful in a suitable medicament choice and proper MPH treatment, especially considering that ADHD is the most commonly reported mental health diagnosis in individuals with prenatal alcohol exposure [58].

It is thought that ADHD in some cases may be genetically inherited, and the theory implies interaction between genetic and environmental factors. For instance, the results of a recently published GWAS meta-analysis confirmed an important role of common variants in the polygenic ethology of ADHD. The authors showed that the loci are located within or near genes that implicate neurodevelopmental processes, likely relevant to ADHD, including *FOXP2*, *SORCS3,* and *DUSP6* [59]. Our analyses not only addressed the impact of genetic factors in the predisposition to ADHD, but also the efficacy and safety of pharmacotherapy, with particular emphasis on drugs affecting dopaminergic and noradrenergic transmission. Dopamine function is widely associated with numerous neuropsychiatric disorders with a probable genetic background; thus, genes encoding dopamine receptors are candidate factors affecting the pharmacological action [60]. No associations were found between *DRD4* polymorphism and clinical outcomes of the MPH treatment. Similarly, no correlation was noted between *DRD4* and other polymorphisms (*HTR1B*, *SLC6A4*, *TPH2*, *DBH*, *ADRA2A*, *COMT,* and *SNAP25*) and MPH response in ADHD patients [61].

The most frequently described polymorphism of the *DRD2* gene is the Taq1A polymorphism (rs1800497), located on chromosome 11, in the proximity of the dopamine D2 receptor gene [62]. The polymorphism constitutes a thymine to cytosine (T/C) substitution at the restriction site for Taq1A. The less frequent A1 allele (minor T allele) is associated with the decreased level of D2 receptor binding which leads to a reduction in the dopaminergic activity [63]. An association of *DRD2* rs1800497 variants with a decreased response to antidepressants has been described, possibly due to reduced dopamine receptor availability for catecholamines [64]. This information may be important in the treatment of depression in patients with FASD, especially in subjects in late adolescence when the dopamine and noradrenergic systems mature, and in older patients, in whom catecholamine availability in the synaptic cleft decreases. For these reasons, our observations may have implications for the safety and efficacy of MPH treatment. Available data indicate that individuals with one copy of the T allele may demonstrate reduced aggression in ADHD, a common symptom of ADHD in comparison to the C/C homozygous genotype, which, in turn, may translate into clinical presentation and, ultimately, the treatment of patients [65]. Another *DRD2* polymorphism (rs1076560) was assessed, among other factors, in terms of the occurrence of psychotic symptoms in chronic cannabis users [66]. It was shown that the T allele of *DRD2* rs1076560 was associated with a threefold greater likelihood of psychotic disorders, compared to the carriers of the GG genotype (OR = 3.07; 95% confidence interval [CI]: 1.22–7.63). Cannabis users carrying the T allele showed a significant impairment in working and detailed memory compared to other genotypes (*p* = 0.008).

Our analysis of the impact of both *DRD2* polymorphisms (rs1076560, rs1800497) on the efficacy and safety of MPH treatment in children with ADHD exposed to high PAE showed no statistically significant differences between the FASD and NFC groups. So far, only a few studies have addressed the influence of *DRD2* polymorphisms on MPH efficacy. Gomez-Sanchez et al. found a significant association between *DRD2* genotypes and MPH response, which contrasts with our results [24]. A faster response effect was observed during the first 3 months of treatment, in patients with the genotypes T/C or C/C, which may partly be related to increased *DRD2* expression and availability in the C allele of rs1800497 carriers [67].

*DRD2* polymorphisms in children with ADHD have also been studied in the context of the relationship between *DRD2* rs1800497 genotypes with food consumption in ADHD children receiving varying doses of MPH [68]. The authors showed an interaction between *DRD2* genotypes and MPH dose. Namely, there was a stronger dose effect in A2/A2 carriers; A2/A2 carriers showed a stronger effect of dose when compared with A1/A1 and A1/A2 children combined (*p* = 0.007). McCracen et al. addressed the importance of DRD1-DRD5 receptor genetic polymorphisms in treating ADHD symptoms in children with autism spectrum disorders [69]. The *DRD2* variant (rs6275) was significantly associated with MPH intolerability; however, there was no influence the response to the treatment. Homozygotes for the common allele at rs6275 showed a sixfold greater rate of intolerance, which may suggest the role of other epigenetic factors in the treatment response.

The A functional polymorphism Val158Met (rs4680) changes *COMT* activity levels. Wild-type Val/Val genotype carriers exhibit four times higher enzymatic activity than Met/Met homozygotes [70]. Similarly to McGough et al., we found no significant *COMT*–MPH dose effects, a significant impact of *COMT* variants, or MPH dose on the ADHD symptom response [71]. However, a few studies suggested a tendency for improved response on hyperactive–impulsive symptoms with an increasing dose of MPH in Val/Val homozygotes [72].

A meta-analysis performed by Myer et al. evaluated the clinically significant predictors of MPH response in children with ADHD [73]. The data based on seven publications revealed that the Val/Val genotype of *COMT* rs4680 was associated with an improved treatment response compared to Met allele carriers (OR: 1.40, CI: 1.04–1.87, *p* = 0.02). Our own research does not confirm those observations, which may be related to the specificity of the studied patients. The analyses performed so far have not distinguished between individuals with ADHD in the course of FASD, which, as already mentioned, might have influenced the final stage of MPH treatment.

The results of our study showed a statistically significant association between the occurrence of adverse effects in children treated with MPH and the presence of the rs4680 *COMT* polymorphism A allele (G > A) (*p* < 0.049). In 7 out of 114 MPH-treated children with ADHD, significant adverse effects were observed, occurring at the time of drug introduction, which did not result in discontinuation of pharmacotherapy. In the group of children with adverse effects, FASD was diagnosed in four children, while in the rest of the group no morphological features of the syndrome were found.

We have observed that the polymorphism in *COMT* rs4680 of A allele of in the hPAE children was involved with strong exhibited adverse effects during MTH treatment. Further observations in healthy non-hPAE samples should be carried out to verify if it is a proper feature for the rs4680 *COMT* A allele variant or an epigenetic role of alcohol in reaction to MPH treatment. The carriers of the low-activity variants of *COMT* in both groups were likely to be intolerant to MPH, whereas the A allele did not affect the treatment efficacy.

Methylphenidate causes a range of adverse effects which are generally acceptable. The most common adverse effects include decreased appetite, sleep disturbances, increased blood pressure, and increased heart rate. The risk of serious adverse effects such as suicidal attempts is very low [74].

We showed that adverse effects of MPH treatment affected less than 7% of ADHD patients. Castells et al. performed a systematic review and meta-analysis of randomized controlled trials comparing MPH with placebo in adults with ADHD. All-cause treatment discontinuation was the primary endpoint. The rate of the treatment discontinuation was greater with MPH than with placebo, but the difference was not statistically significant [OR 1.19, 95% CI: 0.82–1.74, *p* = 0.37, I(2) = 64%] [75]. The study showed that the A allele of *COMT* rs4680, which results in low enzyme activity, was significantly associated with MPH intolerability. This outcome contrasts with the study of Hervas et al. who showed that *COMT* rs4680:G/G (Val/Val) individuals experienced more lasting side effects (*p* = 0.02) in patients with autism spectrum disorder (ASD) [76]. The low-activity Met/Met group had a higher central and peripheral DA level, which may be the reason for the occurrence of adverse effects during the use of MPH [77]. An important obstacle to compare the existing studies with our own results is that a group of patients with ADHD is heterogeneous. Most publications did not take into account the burden of history of PAE and did not distinguish a subgroup of children with FASD, which may be relevant to the symptom profile of ADHD and the treatment response, as was shown in detail above.

So far, several studies have tried to understand the genetic variants underlying interindividual variability in pharmacological parameters, as well as adverse effects and efficacy, however, with inconsistent results. Possible reasons may be small group sizes, different methodologies, or selection of other polymorphisms. Importantly, only a few publications have directly addressed the association of genetic variation with efficacy and safety of MPH therapy for ADHD in children with FASD. Animals with PAE have an exaggerated response to psychostimulants; similarly, children with FASD who have structural or neurochemical changes in the central nervous system are often hypersensitive to side effects of the treatment, which may be further enhanced by a genetic factor [78].

The presented data on the impact of the A (Met158) *COMT* allele implies that in the case of an early onset of MPH adverse effects without achieving a therapeutic effect, especially in children without features of FASD, an attempt should be made to reduce the dose before discontinuation [79].

## 5. Conclusions

Our findings suggest that the analyzed *DRD2* and *COMT* gene polymorphisms do not play a role in methylphenidate efficacy in children with high PAE and ADHD. Low-activity variants of *COMT* (Met158) carriers may be more intolerant to MPH. This result may be of potential importance in the treatment individualization of ADHD children with FASD syndrome.

As was mentioned above, the multiple structural and neurobehavioral consequences in a course of teratogenic hPAE effects are well documented. Some of the brain lesions are clinically common to FASD and ADHD children, especially in an area of cognitive domains, motor hyperactivity, immoderate impulsivity, and social functioning in general. All these symptoms are also typical of the so-called morphological organic changes which are clinically expressed in children by means of emotional and behavioral disturbances coexisting with ADHD symptoms. It is rather difficult to differentiate if they are of an organic or functional background and at the moment of choosing the most proper medicament. The experiment conducted by the authors could have shown rather positive observations that only a portion (about one third in our study) of children who had been exposed early to alcohol poison effects (hPAE) in their gravy manifested ADHD symptoms. Moreover, most of these children were methylphenidate treatment successful (over 90%), especially those with FASD and hyperactivity/impulsivity (over half of them) compared to those with non-FASD who showed a better response in the cognition/impulsivity skills. We might achieve a rather optimistic prognosis for children with a diagnosis of ADHD and probable hPAE interview (as their mothers do not always say the truth or/and typical physical abnormalities including facial dysmorphology may not be clearly manifested); unless there are many alarming data in the beginning (for example the presence of “soft” neurological signs, or early school and behavioral difficulties), the prognosis of their proper development seems to be good, especially when the symptoms tend to be reduced during methylphenidate treatment. Organic factors seem not to be so severe and functional disturbances dominate, which are sensitive to MTH therapy. It is worth pointing out that such children with probable hPAE factors are in greater demand for MTH doses (sometimes 3 times higher than usual in ADHD). Such good and successful MPH therapy for ADHD–FASD coexisting syndrome might give a better prognosis of its course because of dominant dopaminergic immaturity than endogenous (genetic or morphologic/organic) changes. Further observations in a bigger group of children (divided into sex subgroups) with hPAE are going to be continued. The analyzed *DRD2* and *COMT* gene polymorphisms seem to play no role in MPH efficacy in ADHD children with hPAE, while low-activity *COMT* (Met158) variant carriers may be more intolerant to MPH. The MPH treatment is effective in ADHD independent of FASD, although the ADHD-FASD variant requires higher doses to be successful. These results may help in optimization and individualization in child psychiatry.

The epigenetic role of alcohol on the clinical intercourse and possible therapeutic CNS response to catecholamine-involved drugs in children with FASD and *ADHD* features should be further investigated.

The limitations of the present study mostly concern the small sample size, a potential sex bias FASD diagnosis, and exclusivity on morphology, because of other scanty precise methodological tools (lack of neuro-illustration scans, neurochemical changes as vanillylmandelic acid activity, or anthropometric data). The authors are worried that using valid tests such as kiddie–SADS and CGI (National Institute of Mental Health, 1985) is surely more appropriate and precise to compare gathered results with other published data. It is worth mentioning that the time for therapeutic effect observation was too short and often ceased because of added agents, for example the necessity to change the model of conducted therapy (the parents were impatient or irritated and disappointed with the effects, or the pupils had school difficulties because of conduct disorder). Such remarks required verification of the pharmacological form of therapy, and methylphenidate therapy was given up or completed by means of another drug (for example small doses of risperidone). These activities were outside the range of the subject of this paper.

## Figures and Tables

**Table 1:** Demographic data of patients involved in the study.

hPAE Group	*n*	Age [Years]	Female Sex [*n*]	Female Sex [%]
total	303	8.94 ± 4.9	213	70.30%
FC	141	8.96 ± 5.07	84	59.57%
PFC	42	8.60 ± 3.87	37	88.07%
NFC	120	9.03 ± 5.06	92	76.67%

hPAE—high prenatal alcohol exposure, FAS children—FC, partial FAS children—PFC, no FASD children—NFC.

**Table 2:** TaqMan^®^ assays.

SNP	Gene Name	Gene Symbol	SNP Location	Nucleotide Change	TaqMan Assay ID
rs4680	catechol-O-methyltransferase	*COMT*	22q11, coding region	G > A	C__25746809_50
rs1076560	dopamine receptor D2	*DRD* *2*	11q23, intergenic	C > A	C___2278888_10
rs1800497	dopamine receptor D2	*DRD* *2*	11q23, coding region	G > A	C___7486676_10

**Table 3:** ADHD treatment response in FASD and NFC groups.

Symptoms	FASD (*n* = 71)					NFC (*n* = 43)					
	BT	±SD	AT	±SD	AFC *	BT	±SD	AT	±SD	NFC *	AFC/NFC *
I attention deficit	26.3	7.4	24.6	7.0	0.2024	23.7	4.6	15.2	3.5	<0.001	<0.001
II impulsivity	10.3	2.7	7.6	2.2	<0.0001	4.5	0.9	4.2	1.1	0.1274	<0.001
III overactivity	12.0	3.7	11.1	3.7	<0.0001	10.4	2.9	9.0	3.0	0.0163	<0.001
total	48.6	12.0	43.3	10.3	0.001	38.6	11.2	28.4	8.1	<0.0001	<0.001

BT—before treatment; AT—after treatment (number of points counted in each of the three scales, and in the Ist scale max. 27 points is possible, in the IInd 15, in the IIIrd 12); FAS children + partial FAS children = FASD, no FASD children—NFC. AFC—all FASD children; scale by Wolańczyk and Kołakowski on the basis of DSM-IV rating scale (RS) and ICD-10 criteria; SD—standard deviation; * *p*-value, the Mann–Whitney U test.

**Table 4:** Genotype distribution of the studied *COMT* and *DRD2* SNPs in FASD and NFC groups.

Genotype	FASD *n* = 71		NFC *n* = 43		p1	p2	p3	p4
	*n*	(%)	*n*	(%)				
*COMT* rs4680:G > A								
GG	17	(24.0)	6	(14.0)	0.223	0.530	0.235	1.000
AG	40	(56.3)	28	(65.1)				
AA	14	(19.7)	9	(20.9)				
*DRD2* rs1076560:C > A								
CC	46	(64.8)	27	(62.8)	0.688	0.533	0.843	0.526
CA	23	(32.4)	16	(37.2)				
AA	2	(2.8)	0	(0.0)				
*DRD2* rs1800497:G > A								
GG	46	(64.8)	27	(62.8)	1.000	1.000	0.843	1.000
GA	24	(33.8)	15	(34.9)				
AA	1	(1.4)	1	(2.3)				

*p*-values calculated by means of Fisher’s exact test; p1—heterozygotes vs. major homozygotes; p2—minor homozygotes vs. major homozygotes; p3—heterozygotes + minor homozygotes vs. major homozygotes (dominant model); p4—minor homozygotes vs. major homozygotes + heterozygotes (recessive model). FAS children—FC, partial FAS children—PFC, PFC + FC = FASD, no FASD children—NFC.

**Table 5:** Association analysis of *COMT* and *DRD2* SNPs in FASD and NFC groups.

Gene	SNP id	Risk Allele	FASD ^#^	NFC ^#^	*p* *	OR (95% CI) Heterozygotes for a Minor Allele **	OR (95% CI) Homozygotes for a Minor Allele ***	OR (95% CI) Minor Allele Carriers ****
*COMT*	rs4680:G > A	G	0.493	0.535	0.58	0.57 (0.20–1.65)	0.62 (0.18–2.20)	0.58 (0.21–1.64)
*DRD2*	rs1076560:C > A	A	0.196	0.186	1.00	0.88 (0.40–1.96)	-	0.96 (0.44–2.11)
*DRD2*	rs1800497:G > A	A	0.188	0.198	0.86	0.98 (0.44–2.19)	0.61 (0.04–10.22)	0.96 (0.44–2.11)

^#^ MAF—minor allele frequency; * *p*-value for comparison of allele frequency (Fisher’s exact test) ** heterozygotes vs. major homozygotes; *** homozygotes for minor allele vs. major homozygotes; **** homozygotes and heterozygotes for minor allele vs. major homozygotes; FAS children—FC, partial FAS children—PFC, PFC + FC = FASD, no FASD children—NFC.

**Table 6:** Demographic data of patients with MPH medication involved in the study.

**MPH Treatment Group**	***n***	**Age [Years]**	**Female Sex [*n*]**	**Female Sex [%]**
total	114	10.08 ± 3.43	77	67.64%
FC	49	10.55 ± 3.38	27	55.10%
PFC	22	9.91 ± 3.37	18	81.81%
NFC	43	9.65 ± 3.53	32	74.42%
	***n***	**TE (*n* = 104)**	**NTE (*n* = 4)**	**AE (*n* = 7)**
FC	49	42	2	5
PFC	22	20	1	1
NFC	43	42	0	1

hPAE—high prenatal alcohol exposure, FAS children—FC, partial FAS children—PFC, no FASD children—NFC. MPH—methylphenidate, TE—treatment effect, NTE—no treatment effect, AE—adverse effects.

**Table 7:** The relationship between studied polymorphisms *DRD2* rs1076560:C > A and *DRD2* rs1800497:G > A and efficacy during MPH treatment.

		TE	NTE	AE	*p* *
	*n* = 114	104	3	7	
	**MAF/MAC**	***n* alleles**			
*COMT* rs4680 G > A	0.500/118	107	0	11	0.04994
*DRD2* rs1076560 C > A	0.1920/43	43	1	0	0.360
*DRD2* rs1800497 G > A	0.1920/43	43	1	0	0.360

TE—treatment effect, NTE—no treatment effect. AE—adverse effects, MAF—minor allele frequency, MAC—minor allele count, * Fisher’s exact test for TE + NTF vs. AE.

## Data Availability

Not applicable.

## Supplementary Materials

The following supporting information can be downloaded at: https://www.mdpi.com/article/10.3390/ijerph19084479/s1, Questionnaire developed by Wolańczyk and Kołakowski on the basis of DSM-IV Rating Scale (RS) and ICD-10 criteria; Supplementary Table S1. Genotype distribution of the studied *COMT*, and *DRD2* SNPs; Supplementary Table S2. Linkage disequilibria between the studied loci in *DRD2* gene; Supplementary Table S3. Comparison of estimated haplotype frequencies of *DRD2* gene between FASD and NFC groups.
