# Supplementary material for: Influence of COMT (rs4680) and DRD2 (rs1076560, rs1800497) Gene Polymorphisms on Safety and Efficacy of Methylphenidate Treatment in Children with Fetal Alcohol Spectrum Disorders
Source: Int J Environ Res Public Health. 2022 Apr 8;19(8):4479. doi: 10.3390/ijerph19084479 (PMC9031832; doi:10.3390/ijerph19084479)
Supplement: Supplementary file 1 [file ijerph-19-04479-s001.zip › ijerph-1608346-supplementary2.pdf]

Table S1. Genotype distribution of the studied *COMT*, and *DRD2* SNPs.

***COMT* rs4680**	**N**	**%**	***DRD2* rs1076560**	**n**	**%**	***DRD2* rs1800497**	**N**	**%**
**Gentype**			**genotype**			**genotype**		
GG	77	25,41%	CC	206	68,21%	GG	198	65,35%
GA	164	54,13%	CA	86	28,48%	GA	93	30,69%
AA	62	20,46%	AA	10	3,31%	AA	12	3,96%
**Allele**			**allele**			**allele**		
G	318	52,48%	C	498	82,45%	G	489	80,69%
A	288	47,52%	A	106	17,55%	A	117	19,31%

***COMT* rs4680 n=303; HWE p=0,14; MAF 0.4752; *DRD2* rs1076560 n=302; HWE p=0,78; MAF 0.1755; *DRD2* rs1800497 n=303; HWE p=0,80; MAF 0.1931**

Table S2. Linkage disequilibria between the studied loci in *DRD2* gene.

**DRD2**	**rs1076560:C>A**	**rs1800497:G>A**
rs1076560:C>A	r^2↓ D′→	0.952
rs1800497:G>A	0.590	

distance (kbp) – 12.86

Table S3. Comparison of estimated haplotype frequencies of DRD2 gene between FASD and NFC groups.

**haplotype**	**FASD**	**NFC**	**p***
GC	0.784	0.829	0.14
AA	0.183	0.154	0.28
AC	0.028	0.013	0.21

***Fisher Exact Test**
