# Supplementary material for: Influence of COMT (rs4680) and DRD2 (rs1076560, rs1800497) Gene Polymorphisms on Safety and Efficacy of Methylphenidate Treatment in Children with Fetal Alcohol Spectrum Disorders
Source: Int J Environ Res Public Health. 2022 Apr 8;19(8):4479. doi: 10.3390/ijerph19084479 (PMC9031832; doi:10.3390/ijerph19084479)
Supplement: Supplementary file 1 [file ijerph-19-04479-s001.zip › ijerph-1608346-supplementaryl1.pdf]

Influence of COMT (rs4680) and DRD2 (rs1076560, rs1800497) genes polymorphisms on safety and efficacy of methylphenidate treatment in children with fetal alcohol spectrum disorders

Małgorzata Śmiarowska, Bogusław Brzuchalski, Elżbieta Grzywacz, Damian Malinowski, Anna Machoy-Mokrzyńska, Anna Pierzchlińska, Monika Białecka

A structured diagnostic interview questionnaire for attention deficit hyperactivity disorder (ADHD) according to ICD-10 and DSM-IV TR

Name of the child:			
Date of examination:		Assessed period*:	
Medications:			

*FOR THE FIRST EXAMINATION, ASSESS THE 6-MONTH PERIOD REQUIRED FOR DIAGNOSIS, FOR SUBSEQUENT EXAMINATIONS AS NEEDED - "2 MONTHS FROM LAST VISIT", "1 MONTH FROM INTRODUCTION OF MEDICATION". MINIMUM PERIOD ASSESSED IS 2 WEEKS.

ATTENTION DEFICIT DISORDER

1. The child is often unable to maintain sustained and close attention to detail or makes mistakes resulted from inattention at school, work, or other activity.
   yes (3) rather yes (2) probably no (1) no (0)
2. The child often fails to maintain sustained attention on tasks/games.
   yes (3) rather yes (2) probably not (1) no (0)
3. The child often does not seem to hear what is being said to it.
   yes (3) rather yes (2) probably not (1) no (0)
4. The child is often unable to follow consecutive instructions or finish schoolwork, assigned work, or duties at work (but not because of oppositional behavior or misunderstanding of instructions).
   yes (3) rather yes (2) probably not (1) no (0)
5. The child often has difficulty organizing its work or other activities.
   yes (3) rather yes (2) probably not (1) no (0)
6. The child does not like, procrastinates or avoids activities that require prolonged mental effort, such as schoolwork or doing homework.
   yes (3) rather yes (2) probably not (1) no (0)
7. The child often loses things needed for work or other activities, e.g., toys, school supplies, pencils, books, tools.
   yes (3) rather yes (2) probably not (1) no (0)
8. The child is easily distracted by external stimuli.
   yes (3) rather yes (2) probably no (1) no (0)
9. The child is often forgetful during daily activities.
   yes (3) rather yes (2) probably not (1) no (0)

Number of symptoms		Number of symptom scores	

HYPERACTIVITY

1. The child frequently has nervous movements of arms or legs or is unable to sit still, often wiggles arms or legs or fidgets in a chair.
   yes (3) rather yes (2) probably no (1) no (0)
2. The child stands up from its seat during class or in other situations that require sitting still.
   yes (3) rather yes (2) probably not (1) no (0)
3. The child frequently runs around or climbs on furniture in situations where it is disapproved (in adolescents or adults, there may be present only a feeling of distress).
   yes (3) rather yes (2) probably not (1) no (0)
4. The child is often excessively noisy during play and has difficulty playing quietly.
   yes (3) rather yes (2) probably not (1) no (0)
5. The child is often on the move; it "runs around like a light". The child's behavior is characterized by excessive motor activity
   yes (3) rather yes (2) probably not (1) no (0)

Number of symptoms		Number of symptom scores	

IMPULSIVITY

6. The child often rushes out with an answer before the question is fully asked.
   yes (3) rather yes (2) probably no (1) no (0)
7. The child often fails to stand in line or wait for its turn in a game or group situation.
   yes (3) rather yes (2) probably not (1) no (0)
8. The child frequently interrupts or disrupts others (e.g., interferes with others' conversation or play).
   yes (3) rather yes (2) probably not (1) no (0)
9. The child is often excessively talkative.
   yes (3) rather yes (2) probably no (1) no (0)

Number of symptoms		Number of symptom scores	

B. Some of the child's impairing symptoms of attention deficit disorder or attention deficit hyperactivity disorder (hyperactivity, impulsivity) appeared before the child was 7 years old.
yes (3) rather yes (2) probably no (1) no (0)
C. The child's functional impairment caused by these symptoms occurs in two or more situations (e.g., at school and at home).
yes (3) rather yes (2) probably no (1) no (0)
D. A clinically significant impairment in social, occupational, or school (educational) functioning is present.
yes (3) rather yes (2) probably no (1) no (0)
E. The child's symptoms do not occur in the course of persistent developmental disorder, schizophrenia, or other psychoses and cannot be more accurately recognized as symptoms of another mental disorder (e.g., mood, anxiety, dissociative, or abnormal personality disorders).
yes (3) rather yes (2) probably no (1) no (0)

**Diagnosis by ICD-10:**	
**Diagnosis according to DSM- IV:**	

© Wolańczyk, Kołakowski, 2005-2011 Questionnaires may be used for clinical purposes only. Questionnaire to fill out during diagnostic interview.

Diagnostic criteria for Conduct Disorder (CD) and Oppositional Defiant Disorder (ODD) in Children questionnaire according to DSM-IV-TR.

Name of the child:			
Date of examination:		6 months score:	12 months score:
Medication:			

ODD

1. Common or excessive outbursts of anger (in relation to level of development).
yes (3) **rather yes (2)** **probably no (1)** **no (0)**
2. Frequent quarrels with adult.
yes (3) **rather yes (2)** **probably no (1)** **no (0)**
3. Open adult defiance or disrespect to behavior standard.
yes (3) **rather yes (2)** **probably no (1)** **no (0)**
4. Evident mean behavior toward other people.
yes (3) **rather yes (2)** **probably no (1)** **no (0)**
5. Blaming others for own mistakes or misbehavior.
yes (3) **rather yes (2)** **probably no (1)** **no (0)**
6. Oversensitive/ easily offended
yes (3) **rather yes (2)** **probably no (1)** **no (0)**
7. Common anger/ taking offence.
yes (3) **rather yes (2)** **probably no (1)** **no (0)**
8. Common malicious or vengeful behavior.
yes (3) **rather yes (2)** **probably no (1)** **no (0)**

symptoms number:	symptoms score:

CD

a) Mocking/repressing others (e.g. inflict pain, persistent intimidation, inflict suffering, molest)
yes (3) **rather yes (2)** **probably no (1)** **no (0)**
b) Commencing fights (not counting siblings fights).
yes (3) **rather yes (2)** **probably no (1)** **no (0)**
c) At least one use of weapon that can inflict severe harm (e.g. stick, brick, damaged bottle, knife, gun).
yes (3) **rather yes (2)** **probably no (1)** **no (0)**
d) At least one cruelty act toward other people (tie someone, hurt someone, set fire to somebody).
yes (3) **rather yes (2)** **probably no (1)** **no (0)**
e) Cruelty toward animals.
yes (3) **rather yes (2)** **probably no (1)** **no (0)**
f) At least one crime commitment involving confrontation with victim (e.g. extortion, robbery, pickpocket wallet).
yes (3) **rather yes (2)** **probably no (1)** **no (0)**
g) At least one act of forced sexual activity toward other person.
yes (3) **rather yes (2)** **probably no (1)** **no (0)**
h) At least one fire starting (with risk of hurting someone else or intended serious damage infliction).
yes (3) **rather yes (2)** **probably no (1)** **no (0)**
i) At least one intended damage to property (other than starting fire).
yes (3) **rather yes (2)** **probably no (1)** **no (0)**
j) At least one breaking into someone's apartment, car or house.
yes (3) **rather yes (2)** **probably no (1)** **no (0)**
k) Often lying or break promises for profit or to avoid commitment.
yes (3) **rather yes (2)** **probably no (1)** **no (0)**
l) Stealing objects of great value, without involving confrontation with victim; in home or outside home (e.g. shoplifting, forgery, burglary).
yes (3) **rather yes (2)** **probably no (1)** **no (0)**
m) Staying often outside the house for the night, despite parents ban.
yes (3) **rather yes (2)** **probably no (1)** **no (0)**
n) Running away from home or from foster family, at least twice or for more than one night (excluding running away from physical or sexual abuse).
yes (3) **rather yes (2)** **probably no (1)** **no (0)**
o) Notoriously skipping classes (before reaching 13 years old).
yes (3) **rather yes (2)** **probably no (1)** **no (0)**

symptoms number:	symptoms score:

A. Symptoms causing significant clinical impairment in social, educational or professional life.
yes **no**
B. Patient above 18 years old meeting antisocial personality disorder requirements.
yes **no**
C. Disorder meets: antisocial personality, schizophrenia, mania episodes or depression, persistent developmental disorder or hyperkinetic syndrome requirements/criteria.
yes **no**
D. Emotional disturbance criteria fulfilled (drug, depression, compulsions, hypochondria)?
yes **no**

E. Patient fulfilled at least one requirement before reaching 10 years old?
yes = beginning early childhood type   no = beginning at start of puberty type

**Failure to diagnose**	**ODD**	**CD (clinical + ODD)**	**CD**
